# Serum MicroRNAs as Biomarkers for Hepatocellular Carcinoma in Chinese Patients with Chronic Hepatitis B Virus Infection

**DOI:** 10.1371/journal.pone.0028486

**Published:** 2011-12-08

**Authors:** Peng Qi, Shu-qun Cheng, Hao Wang, Nan Li, Yue-feng Chen, Chun-fang Gao

**Affiliations:** 1 Department of Laboratory Medicine, Second Military Medical University, Eastern Hepatobiliary Hospital, Shanghai, China; 2 Department of Oncology Comprehensive Treatment, Second Military Medical University, Eastern Hepatobiliary Hospital, Shanghai, China; 3 Department of Laboratory Medicine, Second Military Medical University, Changzheng Hospital, Shanghai, China; South Texas Veterans Health Care System, United States of America

## Abstract

**Background:**

MicroRNAs (miRNAs) have been shown to anticipate great cancer diagnostic potential. Recently, circulating miRNAs have been reported as promising biomarkers for various pathologic conditions. The objective of this study was to investigate the potential of serum miRNAs as novel biomarkers for hepatocellular carcinoma (HCC).

**Methodology/Principal Findings:**

This study was divided into four phases: (I) Ten candidate serum miRNAs were detected by using real-time RT-PCR, corresponding 10 HCC patients with chronic hepatitis B virus (HBV) infection and 10 age- and sex-matched healthy subjects. (II) Marker validation by real-time RT-PCR on HBV patients with (n = 48) or without HCC (n = 48), and healthy subjects (n = 24). (III) Marker detection by real-time RT-PCR in sera from another 14 HCC patients before and 1 month after surgical resection. (IV) We examined the correlation between the expressions of candidate serum miRNAs with clinical parameters of HCC patients. Although miR-222, miR-223 or miR-21 were significantly up- or down-regulated between HCC patients and healthy controls, no significant difference was observed in the levels of these miRNAs between HBV patients without and with HCC. MiR-122 in serum was significantly higher in HCC patients than healthy controls (p<0.001). More importantly, it was found that the levels of miR-122 were significantly reduced in the post-operative serum samples when compared to the pre-operative samples. Although serum miR-122 was also elevated in HBV patients with HCC comparing with those without HCC, the difference was at the border line (p = 0.043).

**Conclusions/Significance:**

Our results suggest that serum miR-122 might serve as a novel and potential noninvasive biomarker for detection of HCC in healthy subjects, moreover, it might serve as a novel biomarker for liver injury but not specifically for detection of HCC in chronic HBV infection patients.

## Introduction

Hepatocellular carcinoma (HCC) accounts for 90% of primary liver cancers and it represents the third most common cause of death from cancer worldwide, with an increasing incidence expected in the next decades [1]. The major risk factors are chronic viral hepatitis B and C (HBV, HCV), alcohol abuse, primary biliary cirrhosis, xenobiotics, diabetes, non-alcoholic fatty liver disease and genetic disorders like haemochromatosis and α1-antitrypsin deficiency [2], [3]. In China, HCC is the second highest cancer killer since the 1990s [4] and HBV infection is highly endemic. The high mortality rate is due to its detection at late stage with limited therapeutic options. Indeed, the clinical heterogeneity of HCC and the lack of good diagnostic markers and treatment strategies have rendered the disease a major challenge.

The search for biomarkers for the diagnosis of diseases has become a rapidly growing area of clinical research. Ideally, biomarkers should be easily accessible such that they can be sampled non-invasively. Therefore biomarkers that can be sampled from body fluids, such as serum or urine, are particularly desirable. Circulating nucleic acids (CNAs) are extracellular nucleic acids found in cell-free serum, plasma and other body fluids from healthy subjects as well as from patients. The ability to detect and quantitate specific DNA and RNA sequences has opened up the possibility of diagnosis and monitoring of diseases, especially in the field of cancer [5]. Furthermore, in some recent studies it has been suggested a kind of non-coding RNA—microRNA (miRNA), also exist in cell-free serum and plasma, highlighting the field of using CNAs to diagnose cancer.

MiRNAs are a group of tiny RNAs with a fundamental role in the regulation of gene expression. Aberrant expression of several miRNAs was found to be involved in a large variety of neoplasms [6], including HCC [7]–[12]. A relevant and important feature of miRNAs is their remarkable stability. They are known to be well preserved in tissue samples even after years of formalin-fixation and paraffinembedding, and can be efficiently extracted from and quantified in such specimens [13]. Investigation of cancer-specific miRNAs in the circulation is an emerging and exciting field of study. One of the first studies measuring miRNA levels in serum was reported by Lawrie et al. [14], who showed that sera levels of miR-21 were associated with relapse-free survival in patients with diffuse large B-cell lymphoma. Subsequently, circulating miRNAs have been postulated as novel biomarkers for cancer, and other disease processes [15]–[27]. To date, there have been no systematical reports on the role of circulating miRNAs in HCC, and it is not fully understood whether serum miRNAs have a clinicopathological influence in HCC. We hypothesized that levels of specific cancer-associated miRNAs in circulation would differ between HCC patients and chronic HBV infection patients without HCC or healthy individuals. If this hypothesis held truth, it would signify a major breakthrough in HCC management, bringing us ever closer to finding a novel, sensitive, and noninvasive biomarker for this common disease.

The primary aim of this study was to investigate whether cancer-specific miRNAs are detectable and altered in serum of HCC patients compared with age- and sex-matched disease and healthy controls. We also collected serum samples from HCC patients before and after the tumor resection, and these samples were used to determine whether those up-regulated markers in cancer serum were reduced after the tumor resection. Finally, a potential relationship between circulating miRNAs levels and existing clinicopathological features of HCC, such as tumor number, size, growth phase, stage, Child-pugh grade and overall survival, was investigated.

## Results

### Patient Characteristics

A total of 152 participants including 70 HBV-positive HCC patients, 48 chronic HBV infection patients without HCC, and 34 normal subjects were recruited into this study (**Table 1**). There were no significant differences of age (t-test) and sex (Pearson χ^2^ test) between cases and controls. In addition, the HCC group and the other two controls groups had statistically different laboratory results for ALB, T-Bil and ALT (p<0.001).

**Table 1:** Summary of clinical details of subjects used for miRNA analysis.

	Healthy control(n = 34)	HBV patientswithout HCC(n = 48)	HBV patientswith HCC(n = 70)
Age, median, y	38	45	49
Men, n (%)	24 (70.8)	37 (77.1)	55 (78.6)
Laboratory values			
mean (SD)			
Total bilirubin^b^, µmol/L	14.1 (5.3)	14.9 (6.2)	19.2 (10.3)
Albumin^a^, g/L	48.6 (2.9)	47.3 (3.5)	40.1 (3.9)
Alanine aminotransferase^b^, U/L	20.3 (12.2)	26.2 (10.6)	116.6 (257.6)
Tumor number (n = 70) n (%)			
Single			48 (68.6)
Multiple			22 (31.4)
Tumor size (n = 70) n (%)			
<5 cm			17 (24.3)
≥5 cm			53 (75.7)
Tumor grade (n = 65) n (%)			
I and II			14 (21.5)
III and IV			51 (78.5)
Tumor stage (n = 70) n (%)*			
I			8 (11.4)
II			36 (51.4)
III			20 (28.6)
IV			6 (8.6)
Hepatic cirrhosis status (n = 68) n (%)			
Positive			51 (75.0)
Negative			17 (25.0)
Child-pugh grade (n = 70) n (%)			
A			59 (84.3)
B			8 (11.4)
C			3 (4.3)

a , significant difference exists between three cohorts (p<0.001).

b , significant difference exists between HCC group and non-HCC groups (p<0.001).

*Tumor stage was obtained according to the TNM criteria.

With regard to clinicopathologic characteristics of HCC patients, single tumor was found in 48 patients (68.6%), tumor diameter was <5 cm in 17 patients (24.3%), 51 patients were also with hepatic cirrhosis. The histologic grade of HCC was grade I–II in 14 cases, grade III–IV in 51 cases. Tumor stage was obtained according to the TNM criteria, 8, 36 and 20 patients was in stage I, II, III, and only 6 patients was in stage IV. According to the Child classification [28], 59, 8 and 3 patients was with mild (grade A), moderate (grade B) and severe (grade C) liver damage, respectively. All HCC patients had completed follow-up. Forty-one patients who survived more than 20 months (average, 25.65 months; range, 21.3 to 31.3 months) on the last follow-up were classified as the longer-survival group, whereas the rest twenty-nine patients who had survival times less than 20 months (average, 10.93 months; range, 5.3 to 19.2 months) were classified as the shorter-survival group.

### Identification of HCC-associated MiRNAs in Serum

**Figure 1:**
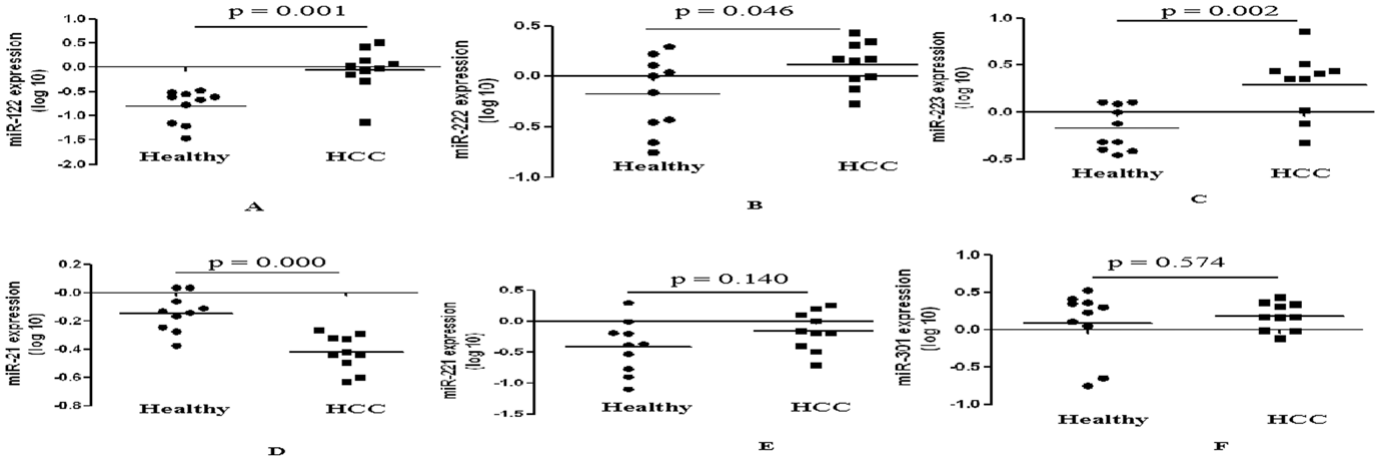
Expression levels of miRNAs in serum of HCC patients and healthy subjects. Levels of miR-122 (A), miR-222 (B), miR-223 (C), miR-21 (D), miR-221 (E), and miR-301 (F) were measured in serum of HCC patients (n = 10) and healthy subjects (n = 10) by RT-qPCR. Using miR-16 as normalization control, 3 significantly up-regulated miRNAs (miR-122, miR-222 and miR-223) and 1 significantly down-regulated miRNA (miR-21) in serum in HCC patients were identified, while expression levels of miR-221 and miR-301 in serum were non-significantly higher in HCC patients than in healthy subjects.

The goal of the present study was to explore the potential use of serum miRNAs as biomarkers for HCC. In this marker discovery phase, a panel of 10 cancer associated miRNAs was chosen on the basis of their reported relevance to HCC [7]–[12] and detected by RT-qPCR among 10 HCC patients and 10 healthy subjects. Using miR-16 as normalization control, 3 significantly up-regulated miRNAs (miR-122, miR-222 and miR-223) and 1 significantly down-regulated miRNA (miR-21) in serum in HCC patients were identified (**Figure 1 A–,B, C, D**), while expression levels of miR-221 and miR-301 in serum were non-significantly higher in HCC patients than in healthy subjects (**Figure 1 E, F**). As for miR-224, let-7a and miR-199a, the detection rates were <50% in serum samples by RT-qPCR, thus, these miRNAs were not chosen in further analytic studies.

### Marker Selection and Validation in Serum Samples

**Figure 2:**
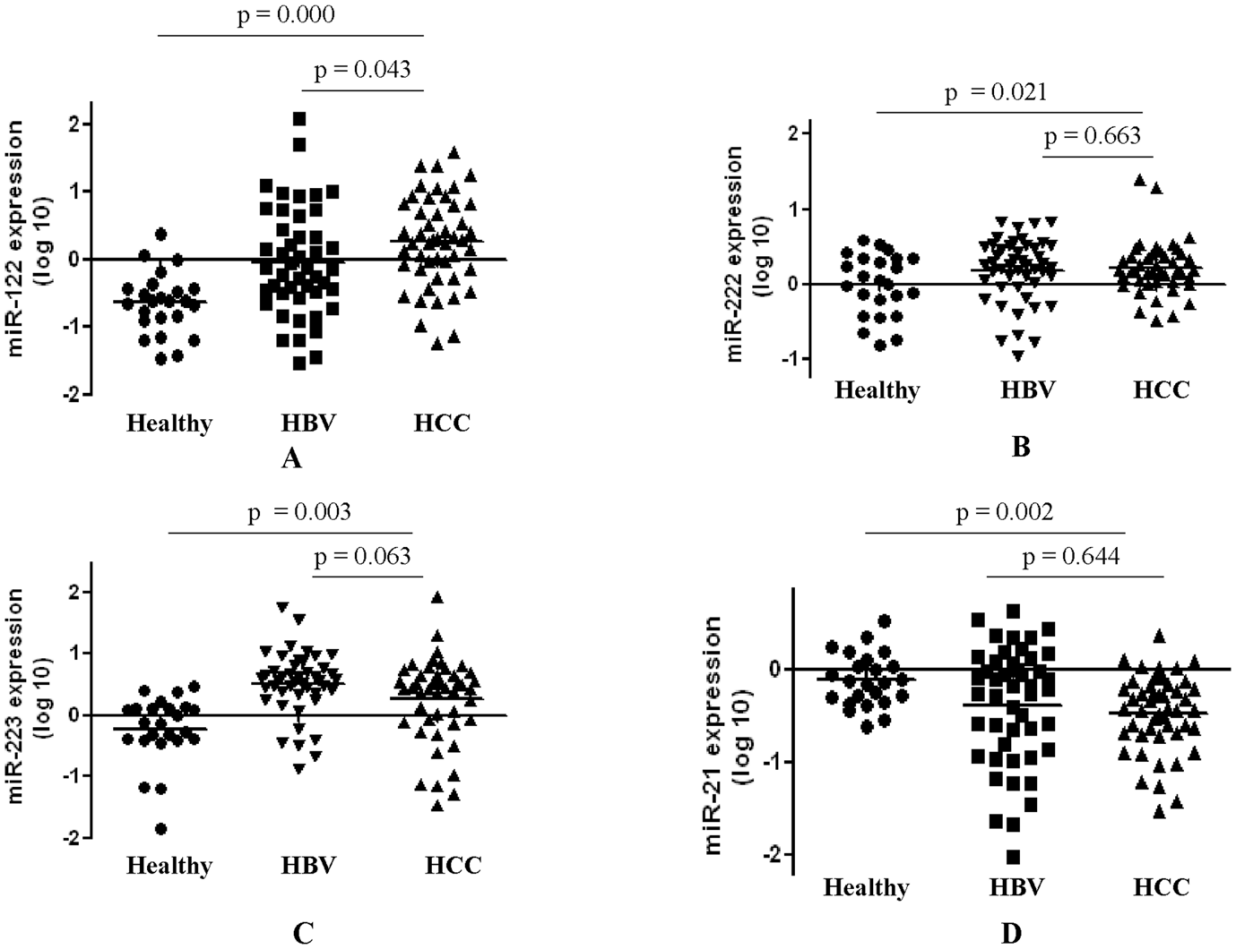
Expression levels of miRNAs in serum of HCC patients, HBV patients without HCC and healthy subjects. Levels of miR-122 (A), miR-222 (B), miR-223 (C) and miR-21 (D) were measured in serum of 48 HCC patients (n = 48), HBV patients without HCC (n = 48) and healthy subjects (n = 24) by RT-qPCR. Levels of miR-122 were significantly higher in HCC patients than disease controls (p<0.05) or healthy controls (p<0.001). Although levels of miR-222 and miR-223 were also significantly elevated in HCC patients than in healthy controls (p<0.05), no significant difference was observed for these two miRNAs between HBV subjects with and without HCC (p>0.05). Levels of miR-21 were reduced in HCC patients than in healthy controls (p<0.05), no significant difference was observed for miR-21 between HBV subjects with and without HCC (p>0.05).

To validate the 4 putative markers identified from the marker discovery phase, RT-qPCR assays were developed to quantify miRNAs in serum among 48 HCC patients, 48 HBV patients without HCC and 24 healthy subjects. Our data indicated that expression levels of miR-122 in serum were significantly higher in HCC patients than disease controls (p<0.05) or healthy controls (p<0.001) (**Figure 2 A**). Although levels of miR-222 and miR-223 were also significantly elevated in HCC patients than in healthy controls (p<0.05), no significant difference was observed for these two miRNAs between HBV subjects with and without HCC (p>0.05) (**Figure 2 B, C**). Interestingly, the levels of miR-223 in serum of HBV patients without HCC were non-significantly higher than those in patients with HCC. In addition, levels of miR-21 were reduced in HCC patients than in healthy controls (p<0.05), no significant difference was observed for miR-21 between HBV subjects with and without HCC (p>0.05) (**Figure 2 D**).

**Figure 3:**
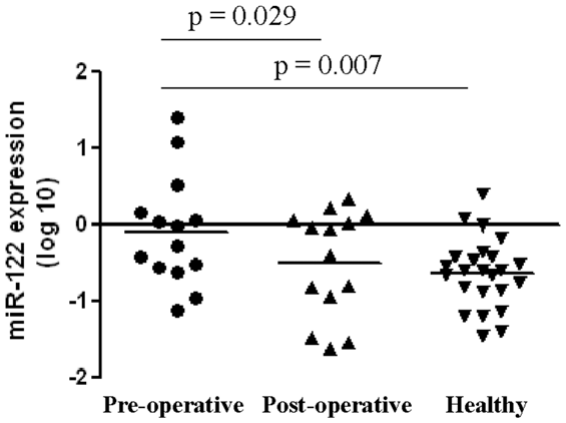
Expression levels of miR-122 in pre-operative, post-operative and healthy serum samples. The levels of miR-122 were significantly reduced in the post-operative serum samples (n = 14) when compared to the pre-operative samples (n = 14), reaching levels comparable with healthy subjects (n = 24).

In order to prove circulating miR-122 in serum is of tumor origin, its levels were measured in an independent set of 14 HCC patients (before and one month after surgical removal of the tumors). It was found that the levels of miR-122 were significantly reduced in the post-operative serum samples when compared to the pre-operative samples, reaching levels comparable with healthy subjects (**Figure 3**).

### The Diagnostic Value of MiR-122 for HCC

**Figure 4:**
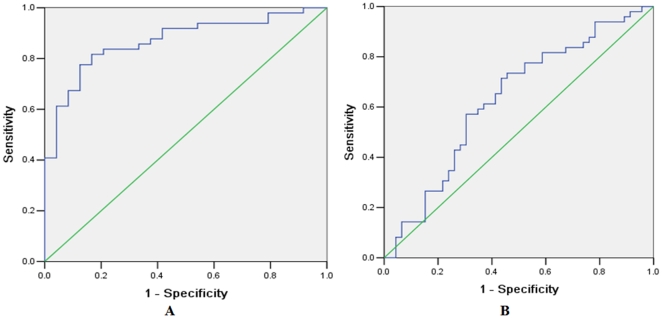
Receiver operating characteristics (ROC) curve analysis using serum miR-122 for discriminating HCC patients from healthy controls and HBV patients without HCC. Serum miR-122 yielded an AUC (the areas under the ROC curve) of 0.869 (95% CI: 0.786–0.952) with 81.6% sensitivity and 83.3% specificity for discriminating HCC patients from healthy controls (A), and an AUC of 0.630 (95% CI: 0.516–0.743) with 77.6% sensitivity and 57.8% specificity for discriminating HBV patients with HCC from those patients without HCC (B).

To evaluate whether serum miR-122 can be used as a potential diagnostic marker for HCC, ROC curve analyses were performed. It was revealed that serum miR-122 was a potential marker for discriminating HCC patients from healthy controls with an AUC (the areas under the ROC curve) of 0.869 (95% CI: 0.786–0.952) (**Figure 4 A**). At the cut-off value of 0.475, the sensitivity and specificity for this marker was 81.6% and 83.3%. However, the AUC of serum miR-122 for discriminating HBV patients with HCC from those without HCC was only 0.630 (95% CI: 0.516–0.743) (**Figure 4 B**). At the cut-off value of 0.651, the sensitivity and specificity for this marker were 77.6% and 57.8%.

### Relationship of Circulating MiRNAs to Clinicopathological Parameters

It was reported that some unique miRNA signatures were associated with prognostic factors and disease progression in several cancers. Therefore, we examined the correlation between the expression of circulating miR-122, miR-222, miR-223 and miR-21 with clinical parameters. No significant association was found between the four miRNAs and tumor number, size, growth phase, stage, Child-pugh grade and overall survival (p>0.05), while the levels of miR-122 demonstrated an elevation trend in patients with small tumor (p = 0.055). In addition, large tumor patients were found to have non-significantly higher levels, on average, of serum miR-21 compared with those with small tumor (p = 0.051). A potential relationship between circulating miRNA levels and status of liver cirrhosis, was also investigated, but no statistically significant difference was identified for any of these parameters.

## Discussion

HCC represents an extremely poor prognostic cancer that remains one of the most common and aggressive human malignancies worldwide. The early diagnosis of HCC is of great clinical desirable and the improved prognosis of HCC if the patients could get surgical treatment early. Up to now, alpha-fetoprotein (α-AFP) has mainly been used in clinic for diagnosis of primary HCC; however, its sensitivity and specificity are not satisfying [29], novel biomarkers for early HCC diagnosis are greatly needed.

Results from recent studies revealed that circulating miRNAs are potential diagnostic biomarkers and prognostic factors in various kinds of diseases, especially in the field of cancer. The first serum miRNA biomarker discovered was miR-21. Lawrie et al. found that patients with diffuse large B cell lymphoma had high serum levels of miR-21, which associated with increased relapse-free survival [14]. Mitchell et al. demonstrated the presence of circulating tumor-derived miRNAs in blood by using a mouse prostate cancer xenograft model system and showed that measurements obtained from plasma were strongly correlated with those obtained from sera, suggesting that both serum and plasma samples would be adequate for measuring specific miRNA levels [16]. In another study, Chen et al. demonstrated that by using serum directly or by extracting RNA from the serum they could identify unique miRNA expression profiles for lung cancer, colorectal cancer and diabetes patients compared with healthy subjects [15]. Circulating miRNAs have also been postulated as novel biomarkers for ovarian cancer [17], [21], pancreatic cancer [19] and colorectal cancer [23], [24]. Although the clinical significance of these findings has not been elucidated in detail, those findings demonstrated that circulating miRNAs could be noninvasive diagnostic or prognostic markers for cancer.

In this study, we confirm that some miRNAs can be measured from a relatively small amount of serum. In addition, as there is no current consensus on the use of house-keeping miRNAs for RT-qPCR analysis, based on previously published results [14], [23] and as recommended by the manufacturer (Applied Biosystems), we used miR-16 levels for normalization. We found that the levels of miR-122 in serum samples from HCC patients were significantly higher than healthy subjects (p<0.001). Although serum miR-122 was also elevated in HBV patients with HCC comparing with those without HCC, the difference was at the border line (p = 0.043). Serum miR-122 yielded an AUC of 0.869 (95% CI: 0.786–0.952) for discriminating HCC from healthy subjects and only 0.630 (95% CI: 0.516–0.743) for discriminating HBV patients with HCC from those without HCC. At a cut-off value of 0.474, the sensitivity was 81.6% and the specificity was 83.3% in discriminating HCC from healthy subjects, and 77.6% sensitivity and 57.8% specificity in discriminating HBV patients with HCC from those without HCC at the cut-off value of 0.651. More importantly, it was found that the levels of miR-122 were significantly reduced in the post-operative serum samples when compared to the pre-operative samples, reaching levels comparable with healthy subjects, indicating that the elevation of serum miR-122 is likely derived from HCC.

MiR-122 not only is evolutionary conserved across species and but also was identified as the most abundant liver specific miRNA constituting 70% of total hepatic miRNAs while cloning small RNAs from different tissues in mice [30], [31]. MiR-122 facilitates replication [32] and translation [33] of hepatitis C viral RNA and positively regulates cholesterol and triglyceride level [34], [35]. Significantly, the down-regulation of miR-122 was detected in more than 70% of HCC [36]. It was shown that the level of miR-122 expression increases in the mouse liver throughout development, to reach the maximum just before birth. Thus, the loss of expression of miR-122 of HCC cells may represent either a differentiation reversion or a block to a less differentiated status of liver cells. In our study, it appears contrary and unexpected that the levels of miR-122 are elevated in serum of HCC patients. Our results showed that the elevated serum miR-122 is presented not only in HBV patients with HCC but also in HBV patients without HCC, suggesting that the elevated miR-122 in the serum of patients may also reflect liver injury [22], [37]. Hepatocytes contain abundant miR-122 and damage of hepatocytes caused by inflammation due to virus infection or cancer would be expected to release significant amount of this miRNA into the circulation. Because serum miRNAs have been shown to be very stable [16], miRNAs leaked from damaged hepatocytes would accumulate in blood to a high level. This might explain why miR-122 is down-regulated in HCC tissues but elevated in serum of HBV patients without or with HCC. Interestingly, our data indicated that expression levels of miR-122 in serum were significantly higher in HCC patients than disease controls or healthy controls, while Xu et al. showed that expression levels of serum miR-122 were significantly higher in HBV patients than HCC or healthy controls [26]. The reason may be that we and Xu et al. use the different normalization control (miR-16 vs miR-181a and miR-181c).

MiR-223 is one of the miRNAs that has been given much attention in the literature. This miRNA is usually regarded as a bone marrow specific miRNA that functions as an important modulator of cellular differentiation [38], [39]. In addition to this, a recent study observed that miR-223 was commonly repressed in HCC [11], suggesting a potential role of this miRNA in liver disease. In our study, levels of miR-223 were significantly elevated in HCC patients than in healthy controls, while no significant difference was observed for this miRNA between HBV subjects with and without HCC. Moreover, the levels of miR-223 in serum of HBV patients without HCC were higher than those in HCC patients or healthy subjects. This finding points out that elevated serum miR-223 could also come from tissue injury such as hepatitis. Since patients with chronic hepatitis may have more serious damage of hepatocytes than patients with HCC, it is reasonable to see much higher level of serum miR-223 in patients with chronic B hepatitis than in patients with HCC. For example, similar results have been obtained in the previous study, showing that elevation of serum miR-223 come from hepatic ischemia/reperfusion injury [40].

MiR-21 is one of the most prominent miRNAs implicated in the genesis and progression of human cancer. The earliest study showed that miR-21 is commonly and markedly up-regulated in human glioblastoma, and inhibition of miR-21 expression leads to caspase activation and associated apoptotic cell death in multiple glioblastoma cell lines [41]. Subsequently, there is a growing body of evidence to prove that miR-21 is overexpressed in a variety of tumors such as breast cancer [41], lung cancer [42], colon cancer [43], [44], and HCC [7], [8] with proproliferative and anti-apoptotic function. Recent studies have shown that serum miR-21 levels are significantly increased in patients with defused large B-cell lymphoma or ovarian cancer [14], [21]. In the present study, however, we found lower levels of serum miR-21 in HBV patients without or with HCC than in healthy controls. The role played by circulating miRNAs is also poorly understood. The detected circulating miRNAs might be derived from dying tumor cells, from tumor cells that have been lysed, from cells infiltrating the lymphomas, from other tissues affected by ongoing diseases, or because the tumor cells actively secrete miRNAs into the surrounding environment. These results indicate that miR-21 can act as either oncogene or tumor suppressor, depending on the targets they regulate and the upstream factors that can regulate dysfunction of miR-21. Clearly, the exact role of miR-21 in cancer needs to be fully investigated in the future.

Although the diagnostic efficiency of serum miR-122 may not be optimal, a panel of serum miRNA markers may improve the sensitivity and specificity of this assay for HCC screening. Patients with increased serum miRNAs might prompt more accurate and specific clinical examinations.

In conclusion, serum miR-122 might serve as a novel and potential biomarker for detection of HCC in healthy subjects. Moreover, it might serve as a novel biomarker for liver injury but not specifically for detection of HCC in chronic HBV infection patients. Our data serve as basis for further investigation, preferably in large prospective studies before miR-122 can be used as a noninvasive screening tool for HCC in routine clinical practice.

## Materials and Methods

### Study Design and Patient Samples

This study was divided into four phases: Phase I (Marker discovery): In this phase, pre-operative sera from 10 HBV-positive HCC patients were collected from Eastern Hepatobiliary Hospital (Shanghai, China). Sera from 10 age- and sex-matched healthy subjects were collected from Changzheng Hospital (Shanghai, China) as the control. A panel of 10 cancer-associated miRNAs was chosen on the basis of their reported relevance to HCC [7]–[12] (**Table 2**). By comparing miRNA expression levels from HCC serum versus normal serum, two differential miRNA expression patterns were established and then compared. Significantly up- or down-regulated miRNAs were identified for further analysis in phase II.

**Table 2:** Candidate miRNAs for investigation in the circulation of HCC patients.

miRNAs of interest	Previous association with HCC
miR-301, miR-224, miR-221	increased expression in tumorous liver tissues compared with paired adjacent non-tumorous liver tissues from HCC patients^10^
miR-21	increased expression in tumorous liver tissues of HCC patients compared with paired adjacent non-tumorous^7^ or benign tumorous^8^ liver tissues
let-7a	increased expression in tumorous liver tissues compared with paired adjacent non-tumorous liver tissues from HCC patients^7^
miR-222	increased expression in tumorous liver tissues of HCC patients compared with paired adjacent non-tumorous^10^ or benign tumorous^8^ liver tissues
miR-122	decreased or increased expression in tumorous liver tissues of HCC patients compared with normal liver^8,9^
miR-199a	decreased expression in tumorous liver tissues compared with paired adjacent non-tumorous liver tissues from HCC patients^12^
miR-223	decreased expression in tumorous liver tissues compared with paired adjacent non-tumorous liver tissues from HCC patients^11^
miR-16	reliable endogenous control for investigating serum miRNA levels in recent studies^16^

Phase II (Marker selection and validation): Pre-operative sera from another 48 HBV-positive HCC patients were collected from Eastern Hepatobiliary Hospital (Shanghai, China). Sera from 48 chronic HBV infection patients without HCC and 24 age- and sex-matched healthy subjects were collected from Changzheng Hospital (Shanghai, China) as disease and healthy controls, respectively. The phase II analysis did not include the data from phase I. Chronic HBV infection was defined as positivity for HBV surface antigen for at least 6 months, positivity for HBV DNA by PCR analysis, and HBV infection-compatible results in a liver biopsy. An ultrasound scan was performed at baseline to exclude HCC. Putative miRNAs markers identified in phase I were verified on these serum from cases and controls.

Phase III: In this phase, sera from another 14 HCC patients were collected before and 1 month after surgical resection. It is hypothesized that those up- or down-regulated miRNAs in HCC should be significantly reduced or elevated after tumor resection.

Phase IV: We examined the correlation between the expressions of candidate serum miRNAs with clinical parameters of HCC patients.

The study protocol was approved by the Ethics Committee of Second Military Medical University and adhered to the tenets of the Declaration of Helsinki. Additionally, informed consent was obtained from participants for the use of their blood in this study. All patients were positive for HBsAg and did not have any other types of liver diseases such as chronic hepatitis C, alcoholic liver diseases, autoimmune liver diseases, or metabolic liver diseases. The diagnosis of HCC was histopathologically confirmed. Data on all subjects were obtained from medical records, pathology reports and personal interviews with the subjects. The data collected include age, gender, serum albumin (ALB) level, total bilirubin level (T-Bil), alanine aminotransferase (ALT) level, overall survival and HCC features such as tumor number, size and growth phase. Clinical stage of HCC was evaluated on the basis of the TNM classification system. The Child-pugh score allowed to categorize HCC patients in Child-pugh grades A, B and C.

### Serum Preparation and RNA Extraction

The blood was centrifuged at 1600 rpm for 5 min and the serum aliquoted into 1.7 ml Eppendorf tubes, followed by a 15 min high speed centrifugation at 12000 rpm to completely remove cell debris, leaving only circulating RNA. For RNA isolation from serum, 250 µl of serum was homogenized in 750 µl of Trizol LS (Invitrogen). Then 200 µl of chloroform was added to the sample and the mixed solution was centrifugated. After an additional chloroform extraction and precipitation with isopropanol, the pellet was washed twice by centrifugation with 70% ethanol. The RNA pellet was dried for 10 min at room temperature and dissolved in 30 µl of diethylpyrocarbonate (DEPC)-treated water. DNase treatment (Qiagen) was carried out to remove any contaminating DNA. The concentration and quality of RNA was measured by UV absorbance at 260 nm and 280 nm (A260/280 ratio) and checked by gel electrophoresis individually. In general, we obtained 600 ng of RNA from 1 ml of serum.

### Reverse Transcription (RT) and Quantitative PCR (qPCR)

RT and qPCR kits made specifically for accurate miRNA analysis (Applied Biosystems) were used to evaluate expression of the chosen miRNAs from serum samples. The 15 µL RT reactions were performed using a TaqMan® microRNA Reverse Transcription Kit (Applied Biosystems, USA) and incubated for 30 min at 16°C, 30 min at 42°C, 5 min at 85°C, and then maintained at 4°C. For real-time PCR, 1.33 µL diluted RT products were mixed with 10 µL of 2× Taqman PCR master mixture (No AmpErase UNG), 1 µL TaqMan MicroRNA Assay and 7.67 µL Nuclease-free water in a final volume of 20 µL according to manufacturer instructions. All reactions were run on the ABI 7300 (Applied Biosystems, USA) using the following conditions: 95°C for 10 min, followed by 40 cycles at 95°C for 15 s, and 60°C for 1 min. Real-time PCR was done in triplicate, including no-template controls. Relative expression of miRNA was calculated using the comparative cycle threshold (CT) (2^−ΔΔCT^) method [45] with miRNA-16 as the endogenous control to normalize the data. The CT is defined as the number of cycles required for the FAM signal to cross the threshold in real-time PCR. ΔCT was calculated by subtracting the CT values of miR-16 from the CT values of the chosen miRNA. ΔΔCT was then calculated by subtracting mean ΔCT of the control samples from ΔCT of tested samples. Fold change of miRNA was calculated by the equation 2^−ΔΔCT^.

### Statistical Analysis

Due to the magnitude and range of relative miRNA expression levels observed, results data were log transformed for analysis. Data are presented as mean ± SD. There was no evidence against normality for the log transformed data as confirmed using the Kolmogorov-Smirnov test. t-test was used to evaluate expression differences of the chosen miRNAs between cases and controls. Receiver operating characteristic (ROC) curves were constructed and the area under the curve (AUC) was calculated to evaluate the specificity and sensitivity of predicting cases and controls. All statistical tests were two-sided, and a probability level of p<0.05 was considered to be statistically significant. Data analysis was done using SPSS 11.0 software (SPSS, Inc.).
